# Protoplast isolation, transient transformation of leaf mesophyll protoplasts and improved *Agrobacterium*-mediated leaf disc infiltration of *Phaseolus vulgaris*: tools for rapid gene expression analysis

**DOI:** 10.1186/s12896-016-0283-8

**Published:** 2016-06-24

**Authors:** Kalpana Nanjareddy, Manoj-Kumar Arthikala, Lourdes Blanco, Elizabeth S. Arellano, Miguel Lara

**Affiliations:** Ciencias Agrogenómicas, Escuela Nacional de Estudios Superiores, Universidad Nacional Autónoma de México (UNAM), León, C.P.37684 Guanajuato Mexico; Instituto de Fisiología Celular, Universidad Nacional Autónoma de México (UNAM), Ciudad Universitaria, Coyoacan, Ciudad de México C.P. 62210 Mexico; Instituto Nacional de Salud Pública, Av. Universidad 655, Col. Santa Maria, Cuernavaca, Morelos 62100 Mexico; Instituto de Biología, Universidad Nacional Autónoma de México (UNAM), Ciudad Universitaria, Coyoacan, Ciudad de México C.P. 04510 Mexico

**Keywords:** *Agrobacterium* infiltration, Gene expression, Overexpression, *Phaseolus vulgaris*, Protoplasts, RNAi, *SnRK1*, Sonication, Transient transformation

## Abstract

**Background:**

*Phaseolus vulgaris* is one of the most extensively studied model legumes in the world. The *P. vulgaris* genome sequence is available; therefore, the need for an efficient and rapid transformation system is more imperative than ever. The functional characterization of *P. vulgaris* genes is impeded chiefly due to the non-amenable nature of *Phaseolus sp.* to stable genetic transformation. Transient transformation systems are convenient and versatile alternatives for rapid gene functional characterization studies. Hence, the present work focuses on standardizing methodologies for protoplast isolation from multiple tissues and transient transformation protocols for rapid gene expression analysis in the recalcitrant grain legume *P. vulgaris*.

**Results:**

Herein, we provide methodologies for the high-throughput isolation of leaf mesophyll-, flower petal-, hypocotyl-, root- and nodule-derived protoplasts from *P. vulgaris*. The highly efficient polyethylene glycol-mannitol magnesium (PEG-MMG)-mediated transformation of leaf mesophyll protoplasts was optimized using a GUS reporter gene. We used the *P. vulgaris* SNF1-related protein kinase 1 (*PvSnRK1*) gene as proof of concept to demonstrate rapid gene functional analysis. An RT-qPCR analysis of protoplasts that had been transformed with *PvSnRK1*-RNAi and *PvSnRK1*-OE vectors showed the significant downregulation and ectopic constitutive expression (overexpression), respectively, of the *PvSnRK1* transcript. We also demonstrated an improved transient transformation approach, sonication-assisted *Agrobacterium*-mediated transformation (SAAT), for the leaf disc infiltration of *P. vulgaris*. Interestingly, this method resulted in a 90 % transformation efficiency and transformed 60–85 % of the cells in a given area of the leaf surface. The constitutive expression of YFP further confirmed the amenability of the system to gene functional characterization studies.

**Conclusions:**

We present simple and efficient methodologies for protoplast isolation from multiple *P. vulgaris* tissues. We also provide a high-efficiency and amenable method for leaf mesophyll transformation for rapid gene functional characterization studies. Furthermore, a modified SAAT leaf disc infiltration approach aids in validating genes and their functions. Together, these methods help to rapidly unravel novel gene functions and are promising tools for *P. vulgaris* research.

**Electronic supplementary material:**

The online version of this article (doi:10.1186/s12896-016-0283-8) contains supplementary material, which is available to authorized users.

## Background

The common bean, *Phaseolus vulgaris*, is an economically important crop that belongs to the family Leguminosae and is the most essential grain legume for direct human consumption in the world; in Latin America alone, this species holds a stake of more than 70 % [1]. Despite having such enormous agroeconomic relevance, this crop suffers from several widespread major diseases and abiotic stresses, which decrease the crop yield [2, 3]. Attempts at transformation and crop improvement programs have been hampered by this species’ notorious recalcitrance to routine *in vitro* regeneration and transformation [4]. Furthermore, unlike other legumes, *P. vulgaris* has serious limitations, such as an unavailability of mutants and a lack of rapid and efficient tools for transformation, preventing this species from being used as a versatile model for legume-related research. The *P. vulgaris* genome sequence is available [5]; therefore, the need for an efficient and rapid transformation system is more imperative than ever. Although some reports have suggested the feasibility of the stable transformation of common bean using a microprojectile bombardment method [6], this option demands vast resources and intensive work with a miniscule yield compared to the bombardment methods of other model crop plants, including cereals [7]. Such a low efficiency makes this method potentially unusable in small-scale laboratories. As an alternative, the hairy root system is the only adoptable technique available to carry out transient gene functional analysis [8]. Nevertheless, this method is a transformation procedure that demands time and it is not eligible for a high-throughput analysis of heterologous gene expression.

Compared to the stable transgenic approach, the use of transient gene expression assays offers an opportunity to rapidly assess the function of a large number of genes by evaluating the transcriptional activity of promoters and the sub-cellular localization of proteins and to investigate cell biology and physiology, cell wall traits, etc. In plant biology research, protoplast transfection is well established and used efficiently in single-cell-based studies. Plant protoplasts have shown reactions similar to those of intact cells to hormones, metabolites, environmental cues and pathogen-derived elicitors, providing a powerful and versatile cell system for the high-throughput dissection of plant signal transduction pathways in many plant species, such as *Arabidopsis* [9–11], maize and rice [12], *Brassica* [13], sunflower [14], *Populus* [15], *Poinsettia* [16] and palm [17]. On the other hand, the available protocols for *P. vulgaris* protoplast isolation from either cell suspension cultures [18] or cotyledonary leaves [19] are not amenable to transfection [20].

In addition, the *Agrobacterium*-mediated leaf disc infiltration method is another transient system that is routinely exploited in functional analyses of genes. Sonication-assisted *Agrobacterium*-mediated transformation (SAAT) involves subjecting plant tissue to a brief period of ultrasound in the presence of *Agrobacterium*. Unlike other transformation methods, this system has the potential to transform several cell layers and, furthermore, is an easy and reliable approach to carry out gene functional characterization studies [10, 21, 22]. The protocol is potentially suitable for a wide variety of molecular studies, including gene regulation, protein localization, tagged protein expression, chromatin immunoprecipitation, protein-protein interactions, bimolecular fluorescence complementation (BiFC), protein stability, etc. The simplicity of the protocol allows it to be used in other crop plants as well.

The present paper describes novel protocols for protoplast isolation from different *P. vulgaris* tissues, such as leaf mesophyll, flower petal, hypocotyl, root and nodule, that could be further used to perform rapid cell biology, physiology, and biochemical assays, among others. This study also presents a highly efficient polyethylene glycol-mannitol magnesium (PEG-MMG)-mediated transformation protocol for *P. vulgaris* leaf mesophyll-derived protoplasts. To validate this method for gene expression studies, we used the *P. vulgaris* SNF1-related protein kinase 1 (*PvSnRK1*) gene [23]. *SnRK* genes are evolutionarily conserved metabolic sensors that undergo activation in response to decreased energy levels in eukaryotes. Plant *SnRK1* is well characterized and shown to regulate the timing of embryo maturation in *Arabidopsis*, sucrose cleavage in potato [24, 25], and pollen development (due to the failure to incorporate sucrose into starch) in barley [26]. *SnRK1* also interacts with ABA-dependent and ABA-independent pathways in legumes [27]. The non-conserved cDNA region and open reading frame (ORF) of *P. vulgaris SnRK1* were cloned individually in RNAi and constitutive expression (overexpression) vectors and transfected into mesophyll-derived protoplasts for the downregulation and overexpression of *PvSnRK1* transcript, respectively. Furthermore, the concept of transient gene expression is met by providing a modified gene transformation approach, the SAAT, for the leaf disc infiltration of *P. vulgaris*. A β-glucuronidase (GUS)-based assay and the constitutive expression of yellow fluorescent protein (YFP) demonstrate the efficiency of *Agrobacterium* infiltration, T-DNA integration and expression.

## Results

### Optimization of protoplast isolation

#### Selection of suitable tissues for protoplast isolation and Agrobacterium leaf disc infiltration

**Fig. 1:**
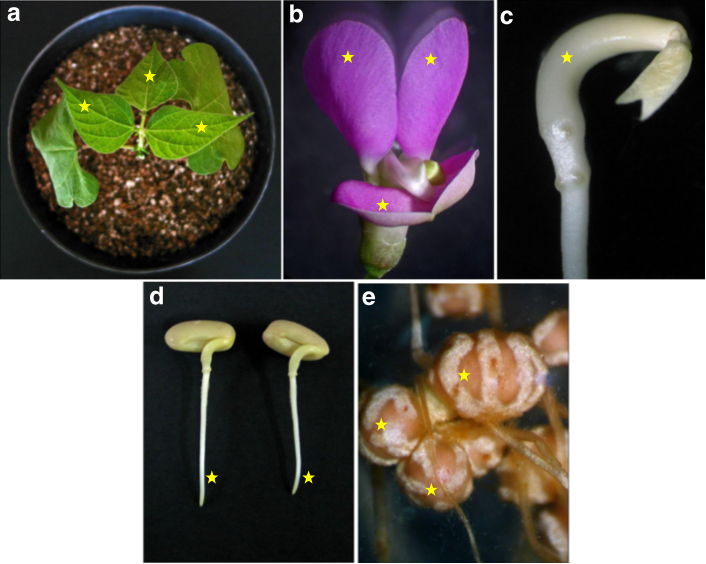
*Phaseolus vulgaris* plant material for protoplast isolation. **a** Ten-day-old wild type plant showing the suitable trifoliate size. **b** Fully bloomed flowers (~40 days after sowing) showing wing and keel petals. **c** Three-day-old decotyledoned germinated seed showing the appropriate stage of the hypocotyl. **d** The root tips of 3-day-old germinated seeds for root protoplast isolation. **e** The mature bean nodule 18–21 days after inoculation with *Rhizobium tropici* strain CIAT 899. Asterisks- the preferable portions of plant material for protoplast isolation

To establish a rapid and suitable system for physiological, biochemical and functional studies of *P. vulgaris*, we aimed to isolate leaf mesophyll protoplasts from the terminal trifoliates of ten-day-old plants (Fig. 1a). Young, healthy and well-irrigated (with B&D nutrient solution) plants that were grown at 28 °C with 65 % humidity were pre-requisites for obtaining intact and uniformly sized protoplasts [10, 18]. Approximately 3- to 6-mm proximal and distal segments of individual leaf blades were removed before slicing the tissues for digestion. To isolate protoplasts from freshly bloomed flowers, the basal segments of standard and wing petals were excised before proceeding to digestion (Additional file 1A). To obtain hypocotyl- and root-derived protoplasts, 1- to 3-day-old and 3- to 4-day-old germinated seedlings (Additional file 1B-C), respectively, were the most appropriate for isolating intact protoplasts. Root tips of approximately 3–4 mm (Additional file 1C) served as good sources of root-derived protoplasts. Choosing the root nodule tissue for isolating *Rhizobium*-infected and uninfected cells was relatively easy, as mature nodules would be undoubtedly be the best source to obtain fully differentiated cells. Hence, nodules 18–21 days post inoculation (dpi) were used in this study (Additional file 1D). SAAT-mediated *Agrobacterium* leaf disc infiltration was highly efficient and successful using the second trifoliates from 10-day-old plants (Fig. 5a). While excising the leaf discs, the midribs were preferentially avoided. However, the size of the disc did not alter the transformation efficiency.

### Isolation of protoplasts

**Fig. 2:**
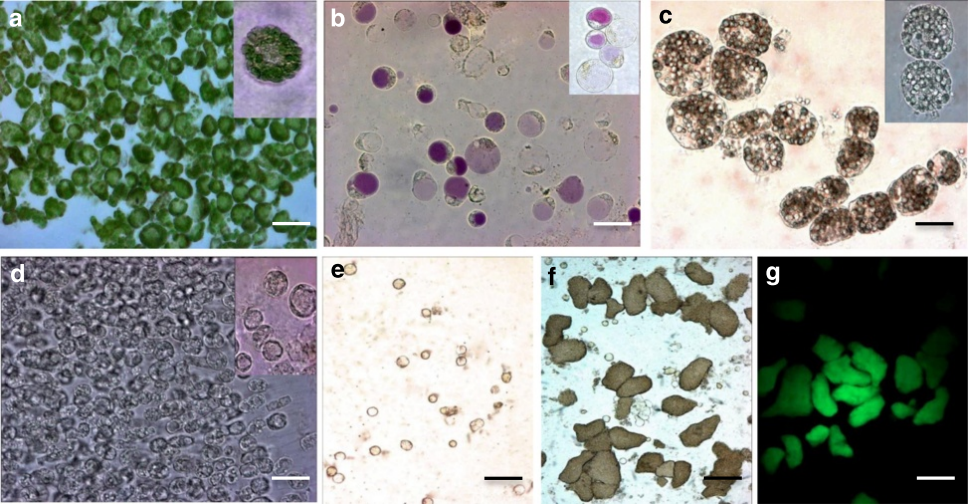
*Phaseolus vulgaris* protoplasts that were isolated from multiple tissues: **a** Leaf mesophyll. **b** Flower petal. **c** Hypocotyl. **d** Root. **e** Uninfected nodule cells. **f** *Rhizobium tropici*-infected nodule cells. **g** *R. tropici* harboring the pSN30-GFP plasmid expressing the GFP protein in infected cells as seen under a laser-scanning confocal microscope. Scale bars: A, D-E, 15 μm; B, 50 μm; and C, F-G, 100 μm

In the present work, we optimized protoplast isolation methods for *P. vulgaris* leaf mesophyll and flower petal with higher yield and efficiency. Table 1 provides a comprehensive overview of the enzyme combinations and conditions for obtaining protoplasts from multiple tissues. To obtain high yields of protoplasts, the leaf tissues were digested with enzyme solution I (ES-I), which contains cellulase and macerozyme. The enzyme concentrations of ES-I were 25 % higher than the concentrations that are used for *Arabidopsis* leaf tissues [10]. The petals were digested with ES-II containing cellulase, macerozyme and pectinase. The tissues were vacuum treated for 30 min to ensure the proper infiltration of the enzyme solution into intercellular spaces to act on the cellulose, hemicelluloses and other cell wall components. A duration of 4–5 h was sufficient to complete the digestion of *P. vulgaris* leaf tissues; however, the petals were completely digested within 8–10 h. High protoplast yields of 3 × 10^5^ cells g^-1^ml^-1^ fresh weight and 2 × 10^5^ cells g^-1^ml^-1^ fresh weight were obtained from leaf mesophyll (Fig. 2a) and petal (Fig. 2b) tissues, respectively.

**Table 1:** Conditions for protoplast isolation from various *Phaseolus vulgaris* tissues

Plant sample	Enzyme solution (ES)		Vacuum infiltration	Plasmolysis	Digestion time	Efficiency
Leaf	ES-I	1.50 % (w/v) cellulase R10, 0.37 % (w/v) macerozyme R10	30 min	N/A	4–5 h	3 × 10^5^ cells g^-1^ml^-1^ FW
Flower petal	ES-II	1.50 % (w/v) cellulase R10, 0.37 % (w/v) macerozyme R10, 30 U pectinase	30 min	N/A	8–10 h	2 × 10^5^ cells g^-1^ml^-1^ FW
Hypocotyl & root	ES-III	2.0 % (w/v) cellulase R10, 0.3 % (w/v) macerozyme R10, 4.0 % (w/v) hemicellulase	N/A	4 h	16–18 h	2 × 10^5^ cells g^-1^ml^-1^ FW
Nodule	ES-IV	1.0 % (w/v) cellulase R10, 0.3 % (w/v) macerozyme R10, 1.0 % (w/v) hemicellulase, 30 U of pectinase	N/A	4 h	16–18 h	1 × 10^5^ cells g^-1^ml^-1^ FW

To digest the hypocotyl, root and nodule tissues, the tissues were first sliced and plasmolysed sequentially in 9 % and 13 % mannitol in CPW (Cell and protoplast washing solution) solution for 2 h each. Then, the plasmolysed hypocotyl and root tissues were treated with ES-III and the nodules tissues with ES-IV (Table 1) for a period of 16–18 h. Interestingly, the mechanical squeezing of the digesting tissues using sterile forceps further increased the protoplast yield. Nevertheless, the prolonged incubation of the samples did not affect the protoplast quality. These protocols were versatile and successful in obtaining high protoplast yields of 2 × 10^5^ cells g^-1^ml^-1^ FW from hypocotyls (Fig. 2c), roots, and nodules (Fig. 2e-g) and 1 × 10^5^ cells g^-1^ml^-1^FW from the root tips (Fig. 2d).

A great variation in protoplast size was observed depending on the source. Hypocotyl-derived protoplasts were the largest (70 to 130 μm), and nodule-uninfected cell-derived protoplasts were the smallest (2 to 4 μm) (Fig. 2e & c). The protoplasts mostly remained spherical, except in case of nodule-infected cells, which were heterogeneously shaped.

### Optimization of the transient transformation of leaf mesophyll-derived protoplasts

**Fig. 3:**
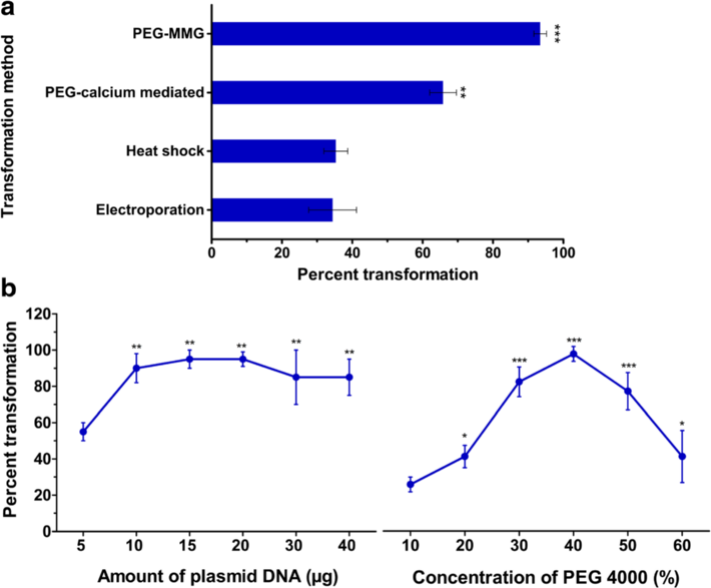
Transformation efficiency of *Phaseolus vulgaris* leaf protoplasts by various transformation methods. **a** All of the experiments were performed using 20 μg of plasmid DNA and 2 × 10^5^ leaf mesophyll protoplasts. The graph shows the percent transformation efficiency in different transformation methods. **b** Percent transformation of protoplasts using various quantities of plasmid DNA (left) and different PEG 4000 (right) concentrations. The statistical significance of differences was determined using a one-way ANOVA Newman-Keuls Multiple Comparison Test (*, *P* < 0.05; **, *P* < 0.01; ***, *P* < 0.001). For both A and B, the data are the averages of three biological replicates (*n* = 9); the error bars represent means ± SD

To further exploit the isolated protoplasts for the functional analysis of genes, we used several methods to transform the leaf mesophyll protoplasts. Transformation approaches, such as electroporation [28, 29], heat shock, PEG mediated transformations, were tested using 20 μg of plasmid DNA (pPZP-RCS-35S/intron GUS) and 2 × 10^5^ leaf mesophyll protoplasts. The protoplasts that were transformed by electroporation showed 35.3 ± 3.4 % transformation efficiency, as determined by GUS staining (Fig. 3a; Additional file 2). Altering several factors, such as electrolytes, electric field and different capacitance for different durations ranging from 10 to 15 s could not significantly improve the transformation efficiency (data not shown). Heat shock also showed a low transformation efficiency ranging from 34.4 ± 6.8 %.

PEG-mediated transformation was performed using two different buffers: 1) PEG-calcium transfection solution: 10–40 % PEG 4000 in distilled water containing 0.2 M mannitol and 100 mM CaCl_2_ [10] and 2) PEG-MMG transfection solution: 10–40 % PEG 4000 in MMG solution [30]. The efficiency was the highest in PEG-mediated transformation in solution with 40 % PEG 4000 (Fig. 3a-b), where 93.4 ± 1.8 % of the protoplasts were transformed by the PEG-MMG method; only 65.8 ± 3.8 % of the protoplasts were transformed in PEG-calcium transfection solution (Fig. 3a & Additional file 4). The concentration of plasmid DNA was the next important factor that influenced the transformation frequency [31]. Utilizing the PEG-MMG transformation approach herein, we analyzed different plasmid DNA quantities to determine the optimal quantity yielding the highest percent transformation. As shown in Fig. 3b, 72 ± 2.1 % transformation was observed using low plasmid DNA quantities, such as 5 and 10 μg. Similarly, high plasmid DNA quantities, i.e., 30 and 40 μg, also resulted in a range of 87 ± 3.1 % transformation. However, 15–20 μg of plasmid DNA was suitable for achieving 92.5 ± 2 % transfection in all of the analyzed constructs (Fig. 3b).

### Gene functional analysis

**Fig. 4:**
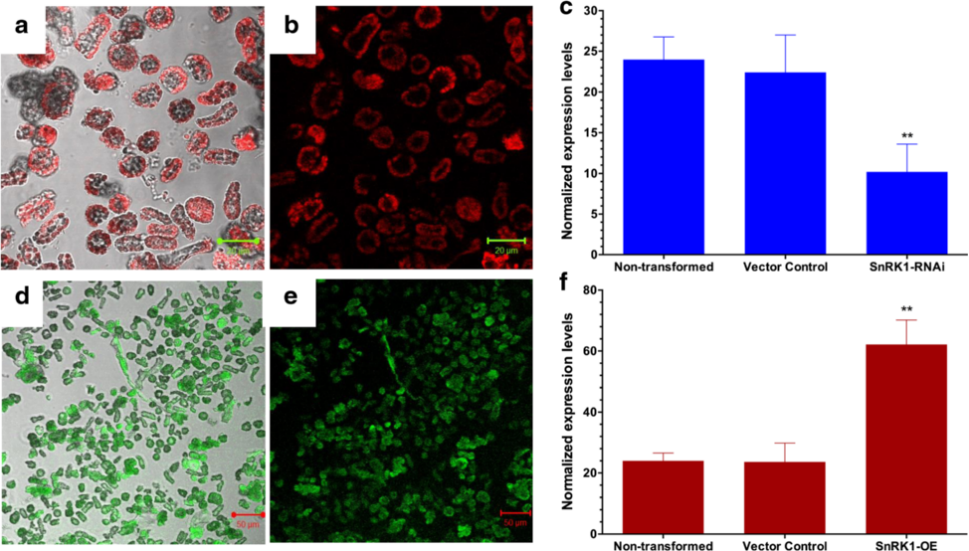
Transformation and gene expression analysis of leaf mesophyll protoplasts of *Phaseolus vulgaris*. **a-b** Laser-scanning confocal microscope showing protoplasts that were transformed with the *PvSnRK1*-RNAi vector expressing red fluorescence. **c** Quantitative RT-PCR analysis showing the downregulation of the *PvSnRK1* transcript in protoplasts that were transformed with the *PvSnRK1*-RNAi vector. **d-e** Protoplasts that were transformed with the *PvSnRK1*-OE vector expressing green fluorescence under a laser scanning confocal microscope. **f** Quantitative RT-PCR analysis showing the overexpression of the *SnRK1* transcript in protoplasts that were transformed with the *PvSnRK1*-OE vector. For RT-qPCR analysis, the total RNA was isolated from transformed protoplasts after 20 h of incubation at room temperature in the presence of light. Transcript accumulation was normalized to the expression of *Ef1α* and *IDE*, which were used as reference genes. The data are the averages of three biological replicates (*n* > 9). The statistical significance of the differences between the control (non-transformed and vector control) and transformed protoplasts was determined using a one-way ANOVA Newman-Keuls Multiple Comparison Test (**, *P* < 0.01). The error bars represent means ± SD

To further examine the feasibility of using *P. vulgaris* leaf mesophyll-derived protoplasts for the functional analysis of the genes, we explored the evolutionarily conserved *SnRK1* gene that is known to regulate energy and stress signaling in eukaryotes. To downregulate the *PvSnRK1* transcript, a plasmid harboring the *PvSnRK1*-RNAi construct was transfected into leaf mesophyll-derived protoplasts. Following 4–6 h of incubation, a fraction of cells was observed under a microscope to verify the expression of red fluorescence protein (RFP) from the transformed protoplasts (Fig. 4a-b). We then determined the percent transformation based on RFP expression; as expected, 92 ± 2.1 % of the protoplasts were transformed successfully. Furthermore, an RT-qPCR analysis of *PvSnRK1*-RNAi transformed protoplasts was performed to validate the downregulation of *PvSnRK1* expression. As depicted in Fig. 4c, the transcripts of *PvSnRK1* significantly decreased by 58 ± 3.2 % in *PvSnRK1*-RNAi-transformed protoplasts compared to those in untransformed and control (transformed with an empty pTdT-RNAi vector) protoplasts. The overexpression of *PvSnRK1* was carried out under the constitutive 35S promoter (pH7WG2D vector, henceforth called ‘*PvSnRK1*-OE’). The transformed protoplasts were selected based on the green fluorescent marker (GFP) (Fig. 4d-e). Furthermore, these protoplasts showed a 91 ± 1.8 % transformation efficiency as determined by GFP-associated fluorescence. RT-qPCR analysis confirmed that *PvSnRK1* transcript accumulation in *PvSnRK1*-OE protoplasts significantly increased relative to that in untransformed and control protoplasts (Fig. 4f). The integration of the RNAi and overexpression constructs in the genome was further confirmed by the PCR amplification of the Tdt fragment (1430 bp) for RNAi and the *PvSnRK1*-35S promoter (460 bp) and GFP (270 bp) for overexpression vectors (Additional file 3) in the transformed protoplasts. Taken together, these results demonstrate the suitability of using *P. vulgaris* mesophyll protoplasts for gene expression studies.

### Transient transformation of *P. vulgaris* leaf disc by an improved SAAT method

**Fig. 5:**
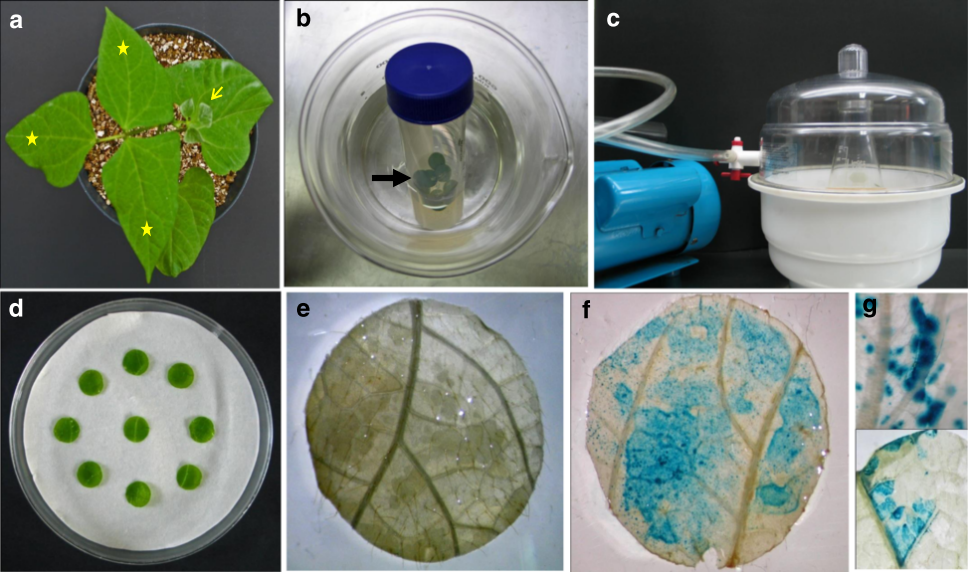
Transient gene expression by the improved SAAT method in *Phaseolus vulgaris* using the pPZP-RCS-GUS binary vector. **a** Ten-day-old plant that was grown in a growth chamber showing the second trifoliates (asterisk) suitable for the transient assay. Arrow- first trifoliate (from shoot apex). **b** The leaf discs in the *vir*-gene-induced *Agrobacterium* culture were first subjected to sonication and **c** later vacuum infiltrated in fresh *Agrobacterium* culture. **d** Co-cultivation of *Agrobacterium*-infected leaf discs on sterile filter paper moistened with MS basal medium. **e** The leaf discs that were transformed with empty vector were stained for the histochemical localization of *β*-glucuronidase (GUS) reporter activity. No blue-stained tissue appeared even after 24 h of incubation with the GUS assay buffer. **f-g** The leaf discs that were transformed with the pPZP-RCS-GUS vector were stained for the histochemical localization of *β*-glucuronidase (GUS) reporter activity. Blue staining appeared within 16 h of incubation

Contrary to the direct transformation approach of mesophyll protoplasts (as demonstrated above), we also attempted to introduce an indirect transformation approach, SAAT [29], for the leaf disc infiltration of *P. vulgaris* (Fig. 5). The leaf disc infiltration assay was carried out using an improved SAAT method utilizing various infiltration media containing a bacterial density of 0.5–0.7 at OD_600_. Among the tested infiltration media, 10 mM MgCl_2_ and a combination of 10 mM MgCl_2_ and 5 mM MES-KOH resulted in 30 and 50 % of transfection efficiencies, respectively (Table 2). Interestingly, a high transfection efficiency of 90 % was observed in Winan’s AB infiltration medium amended with Silwet L-77 (OSi Specialities, Inc., Danbury, CT, USA) and acetosyringone prior to sonication (Table 2) because both Silwet L-77 and acetosyringone are important factors that improve transformation efficiency by increasing the DNA delivery and integration [32]. Microscopic observations of the GUS-stained leaf discs showed that 60–85 % of cells on the leaf surface were transformed by the infiltration medium containing Silwet L-77 and acetosyringone (Fig. 5f).

**Table 2:** Transfection efficiency of *Phaseolus vulgaris* leaf discs that were infiltrated by the SAAT method under different infiltration media

Growth medium	Infiltration medium	Leaf discs*	Transient	Efficiency transfection (%)	Reference
LB	10 mM MgCl_2_	30	9	30	[55]
LB	10 mM MgCl_2_ and 5 mM MES-KOH	30	15	50	[56]
Winan’s AB	Winan’s AB	30	27	90	[54]

*Ten leaf discs were used per biological replicate

**Fig. 6:**
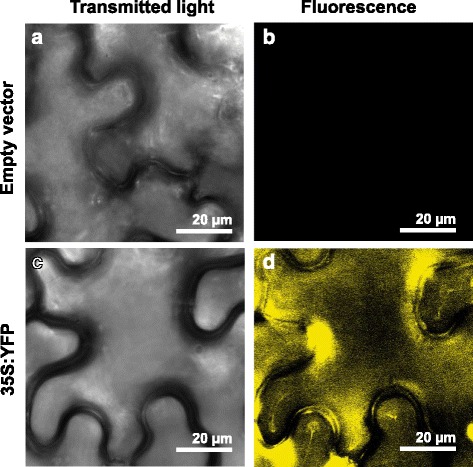
YFP expression vector in leaf epidermal cells of *Phaseolus vulgaris* as delivered through the SAAT method. A representative confocal image showing the empty vector (control) of a pEarleyGate104-transformed leaf under **a** transmitted light and **b** fluorescent light. A representative confocal image showing the expression of the 35S:YFP vector in the leaf epidermis under **c** transmitted light and **d** fluorescent light

To further validate the SAAT transient transformation, we utilized the pEarleyGate104 vector that constitutively expresses yellow fluorescent protein (YFP) in *P. vulgaris* leaf epidermal cells. The confocal images, as expected, showed strong YFP fluorescence in the cytoplasm of leaf epidermal cells (Fig. 6d). We previously showed using the same vector a similar expression pattern of YFP in *P. vulgaris* hairy roots [33]. In contrast, no fluorescence was observed in the leaf epidermal cells that were transformed with empty pEarleyGate104 vector (control) (Fig. 6a-b). Together, these results indicate the suitability of the modified SAAT leaf disc infiltration method to perform gene expression studies and that this method can be extended to studies using a variety of functional analyses, such as gene silencing, protein localization, promoter analysis, etc.

## Discussion

The common bean, *Phaseolus vulgaris,* is a model legume that has been extensively studied worldwide. Given the absence of a reliable protocol for *P. vulgaris* regeneration, the development of an optimal *in vitro* culture system remains a major challenge because this and other species from the *Phaseolus* genus are recalcitrant for *in vitro* regeneration [20]. Thus, the application of biotechnological tools for crop improvement and gene functional characterization in *P. vulgaris* are major limiting factors in plant research. Alternatively, transient expression assays have been indispensable for rapid progress in functional genomics research in other model plants. For instance, the *Arabidopsis* mesophyll protoplast transient expression system is an efficient and useful system for characterizing genes and their functions. However, the use of such a mesophyll protoplast transient system for the model legume *P. vulgaris* is still in its infancy. The optimal plant growth conditions that are associated with highly efficient protoplast isolation, including the optimal enzyme concentration, the length of digestion, the protoplast yield, the percent transfection efficiency, and the use of the system for characterizing exogenously introduced gene(s), have not been previously reported.

The uniqueness of the current study is demonstrated in the procedures that were used to isolate good-quality protoplasts from *Phaseolus* leaves, flower petals, hypocotyls, roots and nodules. The recalcitrant nature of *Phaseolus* often makes it difficult to use *in vitro* suspension cultures. The techniques described here use pot-grown or germinated seedlings as opposed to cells from *in vitro* cultures [18]. Further, there is also an increased possibility that these protoplasts maintain their *in planta* physiology and responses to signal transduction. The key to the successful isolation of protoplasts from various tissues was the plant growth environment because some changes in environmental conditions such as, flooding, extreme temperature, drought and mechanical perturbation will decrease the yield and will also affect the transfection efficiency [10, 34]. The time that was needed for digestion varied depending on the tissue source, and this variation could be due to the changing cell wall composition across tissues. While isolating hypocotyls and nodule protoplasts, the preplasmolysis and osmolarity of the enzyme solution had a significant effect on the protoplast yield [35, 36]. Generally, protoplasts burst in hypotonic solution and collapse in hypertonic solution [37, 38] and in the present study, 9–13 % mannitol imposed appropriate osmotic pressure. The yield and viability of protoplasts are comparable to those of previous reports [39–41].

Thus, isolated protoplasts varied greatly in size and form depending on the tissue source. The protoplasts mostly remained spherical, except in the case of nodule-infected cells, which were heterogeneously shaped, in agreement with previous descriptions of infected protoplasts from determinate nodules [40, 42, 43].

Among the different methods for transfecting leaf mesophyll-derived protoplasts, the PEG-MMG method using 15–20 μg of plasmid DNA (pPZP-RCS-35S/intron GUS) and 40 % PEG was optimal, with 90–95 % transformed cells. This study demonstrates that *Phaseolus* protoplasts could be the system of choice when analyzing gene function either by RNAi or by overexpression. Because protoplasts are non-growing cells, effective RNAi-triggered gene silencing depends not only on the depletion of gene transcripts but also on the turnover rates of corresponding polypeptides. Herein, we tested whether transient RNAi in protoplasts results in the depletion of a targeted polypeptide using the *PvSnRK1*-RNAi vector and also tested the feasibility of the ectopic, constitutive expression of *PvSnRK1* in leaf mesophyll-derived protoplasts. The quantitative RT-PCR results showed that the protoplasts that were transformed with the *PvSnRK1*-RNAi and *PvSnRK1*-OE vectors significantly downregulated and ectopically overexpressed the *PvSnRK1* transcript, respectively. The transfection of RNAi vectors in *Arabidopsis* and rice protoplasts decreases the transcript level of the targeted exogenous and endogenous genes [44, 45].

With the goal of standardizing the methodology for the transient transformation of *P. vulgaris* leaf disc infiltration by SAAT, several aspects were optimized: 1) the density of bacteria required to obtain efficient tissue transformation [30, 46], 2) Winan’s AB medium effectiveness among the different infiltration media [47], 3) the Silwet concentration, and 4) unlike the previous reports, the use of 5 μM acetosyringone in an overnight bacterial culture in Winan’s AB medium and the further addition of 100 μM to the infiltration medium, ensuring highly efficient transformation resulting in approximately 60–85 % of the leaf disc being transformed cells. Furthermore, we validated the system for the constitutive expression of the YFP gene and showed an intense fluorescent protein in the leaf epidermal cells. Thus, these results demonstrate that the modified SAAT leaf disc infiltration method is a simple, highly efficient and rapid process that is suitable for gene expression analysis.

## Conclusions

In this study, we present protocols for the isolation of protoplasts from 5 different tissues of the model legume *P. vulgaris*. We also provide a high-efficiency and amenable method for leaf mesophyll transformation for gene functional characterization studies. Furthermore, we developed a modified SAAT leaf disc infiltration approach that aids in rapidly validating genes and their functions. These methods may help to rapidly unravel the functions of novel genes and represent promising tools for *P. vulgaris* research.

## Methods

### Plant material and growth conditions

In the present study, *Phaseolus vulgaris* L. cv Negro Jamapa was the source of all of the tissue material. The seeds were surface sterilized [8], and 2-day-old seedlings were grown either in sterile vermiculite or on sterile filter paper moistened with Broughton and Dilworth [48] (B&D) nutrient solution in Petri dishes (10 cm diameter) in a growth chamber at 26–28 °C, 65 % humidity. The seedlings were irrigated on alternate days with B&D nutrient solution.

### Developing constructs for protoplast transformation

**Fig. 7:**
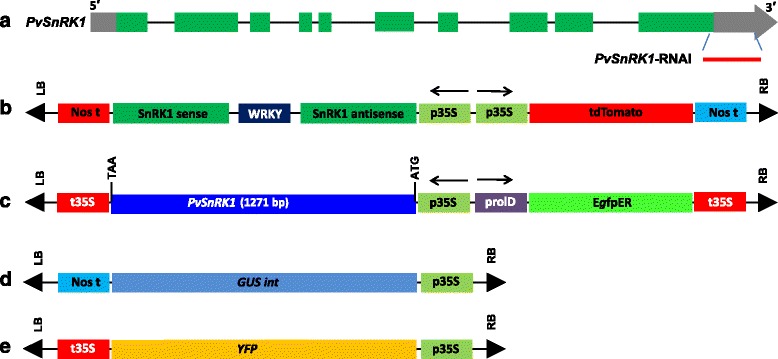
Schematic diagram of the gene structure and T-DNA harboring of *SnRK1* and different the selectable marker genes. **a** Graphic representation of the gene structure of *PvSnRK1* as predicted using the Phytozome v11 (https://phytozome.jgi.doe.gov/pz/portal.html). Green boxes illustrate exons, and gray boxes untranslated regions. The red line represents the non-conserved region of *PvSnRK1* that is used to silence the target gene. **b** A schematic representation of the *PvSnRK1*-RNAi construct showing the sense and antisense region of *PvSnRK1* at the LB. In the opposite orientation, the TdT expression cassette, which encodes a fluorescent selectable marker, is located toward the RB. This construct was carried by the pTdT-DC-*PvSnRK1*-RNAi binary vector. **c** The overexpression of the ORF of *PvSnRK1* under the control of the constitutive 35S CaMV promoter at the LB. In the opposite orientation, the enhanced GFP expression cassette, which encodes a fluorescent selectable marker, is located towards the RB. This construct was carried by the pH7WG2D.1 binary vector. **d** pPZP-RCS binary vector constitutively expressing intron GUS. **e** pEarleyGate104 binary vector constitutively expressing yellow fluorescent protein (YFP). LB, left border; RB, right border; WRKY, hairpin loop; ORF, open reading frame

For gene functional analysis in protoplast transient assays, we utilized the *Phaseolus vulgaris* SNF1-related protein kinase 1 (*PvSnRK1*) (Phvul.008G039400.1) gene (Fig. 7a) to develop RNA interference (RNAi) silencing (downregulating) or overexpression constructs. To generate the RNAi construct, a 209-bp fragment corresponding to the 3′-UTR of *PvSnRK1* (Phvul.008G039400.1) was amplified from cDNA that had been isolated from common bean roots at 2 days post-germination, using the following primers: Forward 5′- CAC CAG ATC TAT GGA CGG ACC AGC TGG CCG-3′ and Reverse 5′- CTC GAG GAG GAC ACG AAG CTG TGC AAG G-3′. The PCR product was recombined with the pTdT-DC-RNAi vector [49] using the Gateway System (Invitrogen) (Fig. 7b).

To develop an overexpression construct of *PvSnRK1*, the ORF of *PvSnRK1* (Phvul.008G039400.1) from *P. vulgaris* cDNA was isolated, and the 1548 bp ORF fragment was inserted into the pH7WG2D.1 binary vector under the control of the constitutive 35S promoter [50] using the Gateway System (Fig. 7c). The correct orientations of the clones were confirmed by sequencing the plasmid insert.

### Leaf mesophyll and flower petal protoplast isolation

**Fig. 8:**
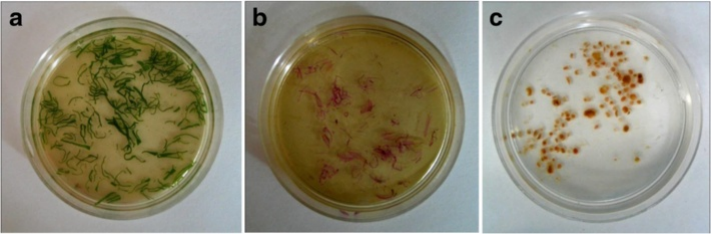
Initial processing of *Phaseolus vulgaris* tissues for protoplast isolation. The plant material was sliced into 0.5–1 mm strips using a sterile scalpel blade and transferred to a Petri dish containing enzyme solution or medium for plasmolysis. **a** Leaf strips in enzyme solution. **b** Strips of wing and keel petals in enzyme solution. **c** Sliced nodules in plasmolysis solution

The first or second trifoliates from the shoot apical tip of ten-day-old plants were used for leaf mesophyll protoplast isolation (Fig. 1a). Standard and wing petals from fully bloomed flowers were used for flower petal protoplast isolation (Fig. 1b). Strips of 0.5–1 mm in thickness were cut from 1 g of both leaf and flower tissues from the portions shown in Additional file 1A, excluding the veins in the leaves. The leaf tissue strips were first vacuum infiltrated for 30 min in enzyme solution I [ES-I; 1.5 % (w/v) cellulase R10 (Yakult pharmaceutical industry) and 0.37 % (w/v) macerozyme R10 (Yakult pharmaceutical industry)] (Fig. 8a) and petal strips in ES-II [ES-I + 30 U pectinase] in 20 mM MES (pH 5.7) with 20 mM KCl, 0.4 M mannitol and 10 mM CaCl_2_ (Fig. 8b). Later, the leaf tissue strips were digested in the dark on a horizontal shaker (40 rpm) at 30 °C for 4–5 h, whereas the flower petals were digested for 8–10 h. The enzymatic reaction was stopped by adding an equal volume of W5 solution [2 mM MES, 154 mM NaCl, 125 mM CaCl_2_ and 5 mM KCl at pH 5.7]. The digested tissue was passed through 108 μm mesh, and the filtrate was collected in a centrifuge tube and incubated on ice for 30 min. Then, the cells were washed twice in W5 solution (10) at 100 g for 3 min each. The protoplast density was calculated using a hemocytometer. Finally, the protoplasts were resuspended in MMG solution (4 mM MES, 0.4 M mannitol and 15 mM MgCl_2_ at pH 5.7) (10) at the desired cell density.

### Hypocotyl and root protoplast isolation

Protoplasts were isolated using the hypocotyl and root tip tissues (Fig. 1c-d) of three-day-old seedlings that were germinated on moistened filter paper. One gram of tissue was used to make 1-mm-thick fragments, which were plasmolysed sequentially in 9 % and 13 % mannitol in CPW solution [(KH_2_PO_4_ (27.2 mg l^-1^), KNO_3_ (100 mg l^-1^), CaCl_2_ (150 mg l^-1^), MgSO_4_ (250 mg l^-1^), Fe_2_(SO_4_)_3._6H_2_O (2.5 mg^-1^l), KI (0.16 mg l^-1^) and CuSO_4_ (0.00025 mg l^-1^) pH 5.8] for 2 h each. The plasmolysed tissue was transferred to ES-III [2 % (w/v) cellulase R10, 0.3 % (w/v) macerozyme R10, and 4 % (w/v) hemicellulase in CPW with 13 % mannitol] and incubated overnight in the dark on a horizontal shaker (40 rpm) at 30 °C. The following day, the tissues were gently squeezed using sterile forceps to facilitate the release of protoplasts. To the solution of protoplasts, an equal volume of W5 solution was added, and the mixture was passed through 108 μm mesh to remove the debris. At this stage, the cells were washed in W5 solution similar to the leaf mesophyll protoplasts and finally re-suspended in MMG solution at the desired hypocotyl (Fig. 2c) and root (Fig. 2d) protoplast density.

### Protoplast isolation from nodules

Mature nodules that were harvested from *P. vulgaris* roots 18–21 days after inoculation with *Rhizobium tropici* expressing the GFP reporter were used for protoplast isolation (Fig. 1e; Additional file 1D). Approximately 500 mg of fresh and healthy nodules (Fig. 1e) was excised from the roots and cut into 0.5–1 mm slices using a sterile razor (Additional file 1D). The nodule slices were plasmolysed in CPW with mannitol (Fig. 8c), similar to the hypocotyl and root tissues. The plasmolysed tissue was transferred to ES-IV [10 mM MES, 0.6 M mannitol, and 1 mM MgCl_2_, with the addition of 1 % (w/v) cellulase R10, 0.3 % (w/v) macerozyme R10, 1 % (w/v) hemicellulase and 30 U of pectinase at pH 5.7] and incubated overnight in the dark on a horizontal shaker (40 rpm) at 30 °C. Later, an equal volume of W5 solution was added to the digested tissue and passed through 108 μm mesh. Uninfected (Fig. 2e) and infected (Fig. 2f-g) cells were further separated by passing the filtrate through 20 μm mesh. At this point, the cells were washed twice in W5 solution by centrifugation at 100 × *g* for 3 min each. Finally, the infected and uninfected cells were re-suspended in MMG solution at the desired cell density.

### Leaf mesophyll protoplast transformation

The pPZP-RCS::GUS [51] binary vector (Fig. 7d) was used to optimize the transformation conditions for *P. vulgaris* leaf mesophyll protoplast studies. The transformation steps were carried out either in a glass Petri plate or glass tube to avoid any loss due to protoplast adhesion. To introduce the plasmid DNA into protoplasts three different approaches such as electroporation, heat shock and PEG mediated transformation were used. Transformation by electroporation (28, 29) was carried out by varying the electrolytes (KCl, CaCl_2_, MgCl_2_), electric field (250 or 300 V cm^-1^) and different capacitance (10, 33 or 50 μF) 2–3 pulses for different durations ranging from 10 s to 15 s with an interval of 20 s. In the heat shock method the protoplasts in CPW13 solution were combined with the plasmid DNA and the solution was exposed to 45 °C for 4–8 min followed by cooling on ice for 10 min.

PEG mediated transformation was carried out employing the same protocol with PEG-CaCl_2_ transfection buffer or PEG-MMG transfection buffers. For PEG mediated transformation, 200 μl (~2 × 10^5^) of protoplasts was pipetted into the center of the Petri plate, 10 μl (10–20 μg) of plasmid DNA (pPZP-RCS-35S-intron GUS) was added, and the plate swirled gently to mix. After incubating at room temperature for 5 min, 200 μl of PEG (40 % PEG 4000 prepared in MMG solution) was added and mixed gently, and the transfection mixture was incubated at room temperature for 15–20 min. The transfection mixture was diluted by adding 2 ml of 0.45 M mannitol at 2 min intervals until the total volume reached to 12 ml. The mixture was mixed carefully after every addition of mannitol. The transfection mixture was transferred to a suitable round bottom glass tube, and the protoplasts were pelleted at 100 g for 3 min. The cells were re-suspended in 1 ml of WI solution [4 mM MES containing 0.5 M mannitol and 20 mM KCl at pH 5.7], transferred to 6- or 12-well tissue culture plates and incubated for 3–6 h under light at room temperature. To stain the transformed protoplast for GUS histochemical activity, the protoplasts were resuspended in GUS reaction buffer and incubated in the dark at 37 °C for 16–24 h according to Jefferson [38]. The GUS-stained cells were mounted with 40 % glycerol in PBS (137 mM NaCl, 2.7 mM KCl, 4.3 mM Na_2_HPO_4_, and 1.47 mM KH_2_PO_4_) and observed under a microscope to assess the percent transformation. Gene functional analyses, such as the downregulation and overexpression of *PvSnRK1,* were carried out using *PvSnRK1*-RNAi and *PvSnRK1*-OE binary vectors, respectively.

### RT-qPCR analysis

The total RNA was isolated from frozen leaf mesophyll-derived protoplasts using the RNeasy Plant Mini Kit according to the manufacturer’s recommendations (Qiagen, USA). Genomic DNA contamination in RNA samples was eliminated by incubating the samples with RNase-free DNase (1 U μl^–^^1^) at 37 °C for 15 min and then at 65 °C for 10 min. The RNA integrity and concentration were determined by electrophoresis and a Nanodrop ND-2000 spectrophotometer (Thermo Scientifics), respectively. For qRT-PCR, 2 μg of total RNA was used to synthesize cDNA.

Quantitative real-time PCR was performed using the iScript^TM^ One-step RT-PCR Kit with SYBR® Green, following the manufacturer’s instructions, in an iQ5 Multicolor Real-time PCR Detection System (Bio-Rad). Each reaction was set up using 40 ng of RNA as the template. A control sample that lacked reverse transcriptase (RT) was included to confirm the absence of contaminant DNA. The relative gene expression levels were calculated using the 2^-∆CT^ method, with ∆CT = CTgene – CTreference gene. *P. vulgaris EF1α* and *IDE* were used as internal controls, as previously described [53, 54]. The relative expression values, normalized with two reference genes, were calculated as previously described [55]. The data are averages of two or three biological replicates, and each sample was assessed in triplicate. The expression of the genes that are listed in Additional file 5 was quantified using gene-specific oligonucleotides.

### *Agrobacterium-*mediated leaf disc infiltration with sonication

*Agrobacterium tumefaciens* strain AGL1 carrying the binary vector pPZP-RCS::GUS or pEarleyGate104 (Fig. 7e) was used for the *P. vulgaris* leaf disc infiltration experiments. Different infiltration media, such as (i) 10 mM MgCl_2_ [55], (ii) 10 mM MgCl_2_ and 5 mM MES-KOH (pH 5.6) [56] and (iii) Winan’s AB medium (pH 5.6) [52] were used to test the SAAT method for *P. vulgaris* leaf disc transformation.

The Agrobacteria were grown on LB agar plates with the appropriate antibiotics for 16–18 h, after which a single colony from the plate was used to inoculate LB broth and further grown for 18 h at 28 °C. An aliquot of 3 ml from the overnight culture was used to inoculate 100 ml of freshly prepared infiltration media, including 10 mM MgCl_2_, 10 mM MgCl_2_ and 5 mM MES-KOH, or Winan’s AB minimal medium amended with the appropriate antibiotics and 5 μM acetosyringone. The culture was further grown for 18 h on incubator shaker (230 rpm) at 28 °C. The OD_600_ was adjusted to 0.5–0.7 with the appropriate media, and SAAT was performed. In the case of Winan’s AB medium, the bacterial culture was divided into two halves, and 10 μl liter^-1^ Silwet L-77 (surfactant; Vac-In-Stuff, Lehle Seeds, USA) and 100 μM acetosyringone were added (each to only one half of the culture). Bean leaf discs (6–11 mm) were immersed in one half of the bacterial culture, sonicated for 5 min and later transferred to the second half of the culture, followed by vacuum infiltration. Vacuum infiltration was carried out for 20–25 min with 2–3 abrupt breaks. Finally, the leaf discs were incubated in the same bacterial culture in the dark for 30 min at 28 °C on a horizontal shaker (40 rpm). Following incubation, the leaf discs were washed 3–4 times in PBS and incubated for 24 h on moistened sterile filter paper towels at 28 °C. Finally, the leaf discs were washed with PBS containing 250 μg ml^-1^ cefotaxime to remove the *Agrobacterium* and were incubated for another 24 h on wet paper towels at 28 °C before further analysis.

### GUS histochemical assay and microscopy

A GUS assay was performed according to Jefferson [38] by incubating the leaf mesophyll-derived protoplasts or *Agrobacterium*-infiltrated leaf discs in the dark at 37 °C for 16–24 h. The β-glucuronidase activity was observed with a brightfield Zeiss Axioplan microscope equipped with DIC optics. Transformed leaf protoplasts expressing TdT (red) and GFP (green) fluorescence were mounted onto slides in 40 % glycerol in PBS (pH 7.4) and observed on a ZEISS-LSM/510 confocal laser-scanning microscope. GFP and YFP fluorescence was excited with a blue argon ion laser (488 nm), and the emitted fluorescence was collected from 510 to 540 nm. RFP fluorescence was excited at 561 nm by a solid-state laser, and emission was filtered using a band-pass filter of 640/50 nm.

## Abbreviations

Dpi, Days post inoculation; ES, Enzyme solution; GFP, Green fluorescent protein; GUS, β-glucuronidase; RFP, Red fluorescent protein; SAAT, Sonication-assisted *Agrobacterium*-mediated transformation; *SnRK1*, SNF1-related protein kinase 1; TdT, tandem dimer Tomato/tomato double tandem

## Additional files

Additional file 1: Selection of appropriate *Phaseolus vulgaris* tissue material for protoplast isolation. (**A**) Wing and keel petals were excised, retaining the pink-colored portions, for protoplast isolation. (**B**) The roots of 3-day-old germinated seeds were cut 3 mm from tip and were used for protoplast isolation. (**C**) Three-day-old germinating seeds were decotyledoned, and ~10 mm hypocotyls were used to isolate protoplasts. (**D**) Sliced 18-dpi nodule that was inoculated with *R. tropici* harboring the pSN30-GFP plasmid expressing GFP fluorescence protein as seen under a laser scanning confocal microscope. Hc, hypocotyl; dpi, days post inoculation; dashed line, site of excision. (DOC 576 kb) Additional file 2: pPZP-RCS-GUS vector-transformed leaf mesophyll protoplasts showing intense GUS expression. GUS staining could be detected within 16 h of incubation with GUS assay buffer. Scale bar: 20 μm. (DOC 181 kb) Additional file 3: PCR-based detection of transgene integration in transformed protoplasts with *PvSnRK1*-RNAi or *PvSnRK1*-35S vector. To evaluate *PvSnRK1*-RNAi and *PvSnRK1*-35S vectors, oligos that were specific to ‘Tdt’ and ‘gene-specific-p35S promoter and GFP’ were used, respectively. gDNA that was isolated form transformed and untransformed leaf mesophyll protoplasts. Lane 1, Tdt; 2, *SnRK1*-35S; 3, GFP; M, molecular weight marker (1 kb); 4-6 are respective untransformed controls for 1-3. (DOC 56 kb) Additional file 4: Percent transformation range and viability of mesophyll protoplast in various transformation methods. (DOC 33 kb) Additional file 5: Primer sequences of *Phaseolus vulgaris* genes used to generate constructs and perform quantitative RT-PCR. (DOC 41 kb)
