# Supplementary figures and images for: Protoplast isolation, transient transformation of leaf mesophyll protoplasts and improved Agrobacterium-mediated leaf disc infiltration of Phaseolus vulgaris: tools for rapid gene expression analysis
Source: BMC Biotechnol. 2016 Jun 24;16:53. doi: 10.1186/s12896-016-0283-8 (PMC4919892; doi:10.1186/s12896-016-0283-8)

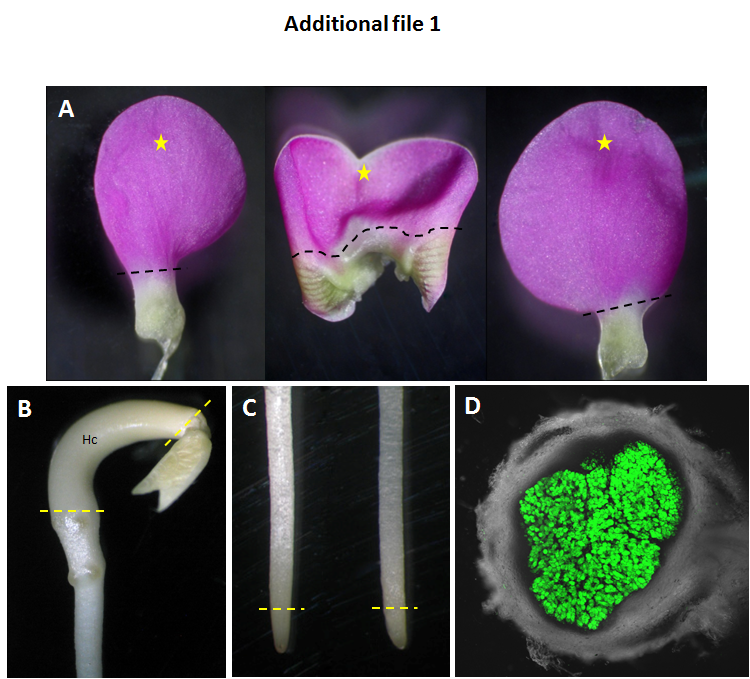

Supplement: Additional file 1: — Selection of appropriate Phaseolus vulgaris tissue material for protoplast isolation. (A) Wing and keel petals were excised, retaining the pink-colored portions, for protoplast isolation. (B) The roots of 3-day-old germinated seeds were cut 3 mm from tip and were used for protoplast isolation. (C) Three-day-old germinating seeds were decotyledoned, and ~10 mm hypocotyls were used to isolate protoplasts. (D) Sliced 18-dpi nodule that was inoculated with R. tropici harboring the pSN30-GFP plasmid expressing GFP fluorescence protein as seen under a laser scanning confocal microscope. Hc, hypocotyl; dpi, days post inoculation; dashed line, site of excision. (DOC 576 kb) [file 12896_2016_283_MOESM1_ESM.doc]

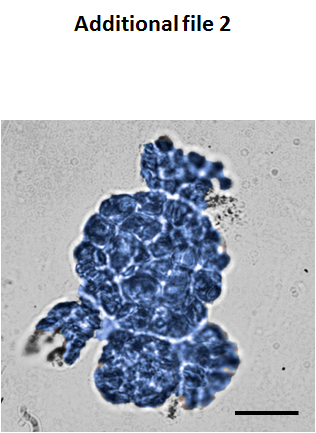

Supplement: Additional file 2: — pPZP-RCS-GUS vector-transformed leaf mesophyll protoplasts showing intense GUS expression. GUS staining could be detected within 16 h of incubation with GUS assay buffer. Scale bar: 20 μm. (DOC 181 kb) [file 12896_2016_283_MOESM2_ESM.doc]

**Additional file 3**

**
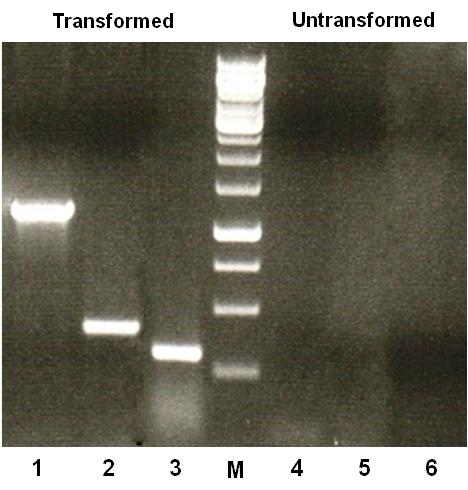
**

Supplement: Additional file 3: — PCR-based detection of transgene integration in transformed protoplasts with PvSnRK1-RNAi or PvSnRK1-35S vector. To evaluate PvSnRK1-RNAi and PvSnRK1-35S vectors, oligos that were specific to ‘Tdt’ and ‘gene-specific-p35S promoter and GFP’ were used, respectively. gDNA that was isolated form transformed and untransformed leaf mesophyll protoplasts. Lane 1, Tdt; 2, SnRK1-35S; 3, GFP; M, molecular weight marker (1 kb); 4-6 are respective untransformed controls for 1-3. (DOC 56 kb) [file 12896_2016_283_MOESM3_ESM.doc]
