# Supplementary material for: Protoplast isolation, transient transformation of leaf mesophyll protoplasts and improved Agrobacterium-mediated leaf disc infiltration of Phaseolus vulgaris: tools for rapid gene expression analysis
Source: BMC Biotechnol. 2016 Jun 24;16:53. doi: 10.1186/s12896-016-0283-8 (PMC4919892; doi:10.1186/s12896-016-0283-8)
Supplement: Additional file 4: — Percent transformation range and viability of mesophyll protoplast in various transformation methods. (DOC 33 kb) [file 12896_2016_283_MOESM4_ESM.doc]

Additional file 4: Percent transformation range and viability of mesophyll protoplast in various transformation methods.

| **Transformation method** | **Transformation range (%)** | **Viability (hours)** |
| --- | --- | --- |
| PEG-MMG | 93.4±1.8 ^a^ | 72±2 ^a^ |
| PEG-calcium mediated | 65.8±3.8 ^b^ | 72±2 ^a^ |
| Heat shock | 35.3±3.4 ^c^ | 31±5 ^b^ |
| Electroporation | 34.4±6.8 ^c^ | 25±2 ^b^ |

The statistical significance of differences among different methods was determined using one-way ANOVA- Newman-Keuls Multiple Comparison Test. In each column, means ± SD followed by different letters indicate statistically significant at p > 0.05.
