# Supplementary material for: Protoplast isolation, transient transformation of leaf mesophyll protoplasts and improved Agrobacterium-mediated leaf disc infiltration of Phaseolus vulgaris: tools for rapid gene expression analysis
Source: BMC Biotechnol. 2016 Jun 24;16:53. doi: 10.1186/s12896-016-0283-8 (PMC4919892; doi:10.1186/s12896-016-0283-8)
Supplement: Additional file 5: — Primer sequences of Phaseolus vulgaris genes used to generate constructs and perform quantitative RT-PCR. (DOC 41 kb) [file 12896_2016_283_MOESM5_ESM.doc]

Additional file 5: Primer sequences of Phaseolus vulgaris genes used to generate constructs and perform quantitative RT-PCR.

| **Gene** | | **Oligonucleotide sequence** |
| --- | --- | --- |
| ***PvEf1α*** | F | 5′-GGTCATTGGTCATGTCGACTCTGG-3′ |
|  | R | 5′-GCACCCAGGCATACTTGAATGACC-3′ |
| ***PvIDE*** | F | 5′-GCAACCAACCTTTCATCAGC-3′ |
|  | R | 5′-AGAAATGCCTCAACCCTTTG-3′ |
| ***PvSnRK1*-RNAi** | F | 5′-CACCAGAGCAAAGAGCTTAGGATCACAAGAGA-3′ |
|  | R | 5′-CTAACTTTTGCAGTATATCTCTTGTTATCC -3′ |
| ***Pv PvSnRK1*-OE** | F | 5′-ATGTCTTCATCAATCTCGTTTCCCACG-3′ |
|  | R | 5′-ACGCAACCGTGTTCCCAAAAACTTTGTC-3′ |
| ***PvSnRK1*** | F | 5′-GAGATTGGGAATGTTTTCATCA-3′ |
|  | R | 5′-GTTATCTCTGGGAAATTGTATGCTG-3′ |
| ***PvSnRK1-35S*** | F | 5′- ATC TCT GGT GTG GAG TAC TGT CAC-3′ |
|  | R | 5′- CCA CTA TCC TTC GCA AGA CCC TTC C-3′ |
| ***Egfp*** | F | 5′- ACC GGC AAG CTG CCC GTG-3′ |
|  | R | 5′- TGC CCC AGG ATG TTG CCG TCC-3′ |
| ***TdT*** | F | 5′- ATG GTG AGC AAG GGC GAG G-3′ |
|  | R | 5′- TTA CTT GTA CAG CTC GTC CAT G-3′ |

F, forward primer; R, reverse primer.
